# Supplementary material for: Nanotopographic control of actin waves and growth cone navigation in developing neurons
Source: Front Cell Dev Biol. 2025 Sep 10;13:1631520. doi: 10.3389/fcell.2025.1631520 (PMC12457675; doi:10.3389/fcell.2025.1631520)
Supplement: Supplementary file 2 [file DataSheet1.pdf]

Supplementary data.

1. Figures:

a.

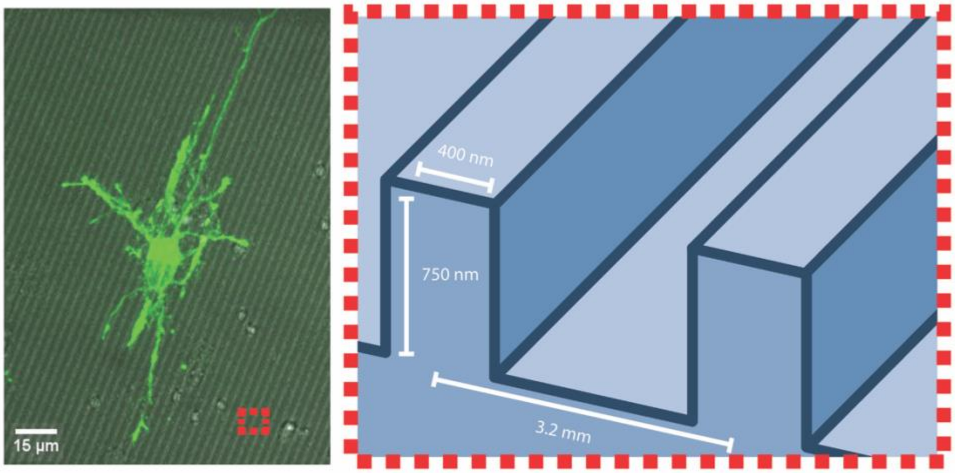

b.

Metric	Early → Middle → Flat (Flat substrate)	Early → Middle (Nanoridges)
Wave speed	no change	↓
Wave velocity	no change	no change
Track displacement	no change	no change
Track length	no change	no change
Track duration	no change	no change
Sinuosity	no change	no change
Track alignment wrt ridges	n/a	↓
Parallel growth cones	n/a	no change
Perpendicular growth cones	n/a	↑

Metric	Flat → Nanoridges
Normalized number of tracks	↓
Recurrence	↓

Suppl. Fig. 1. a. Schematic illustration of the nanoridge dimensions (not to scale), along with a pseudocolored image of a representative DIV 8 primary embryonic cortical neuron transduced

with actin green fluorescent protein and cultured on nanoridges. Approximate sizes of different neuronal structures are indicated: the cell body is approximately 10 μm in diameter, dendrites range from 1 to 8 μm in width depending on their distance from the soma, axons are approximately 1 to 2 μm wide, and growth cones range from 2 to 8 micrometers in width.

b. Results summary showing how actin track metrics change across developmental stages and how these changes are influenced by substrate type, comparing flat surfaces and nanoridges.

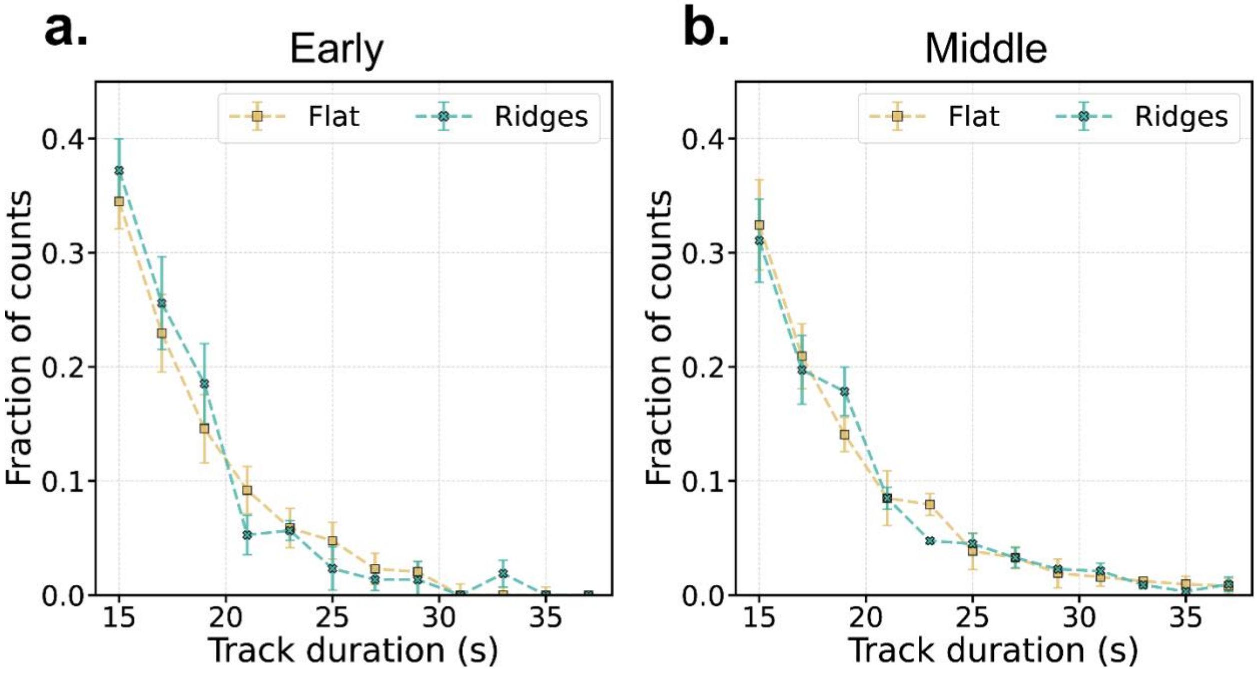

Suppl. Fig. 2. Non-cumulative histograms of above-threshold track durations at the (a) early and (b) middle developmental stages. Distributions are computed using N = 20 bins. Error bars represent the interquartile range (IQR) of the probability density function (PDF) at each bin across all videos for each condition, while markers indicate the corresponding median values. The early stage includes 17 flat and 9 ridge movies; the middle stage includes 21 flat and 6 ridge movies.

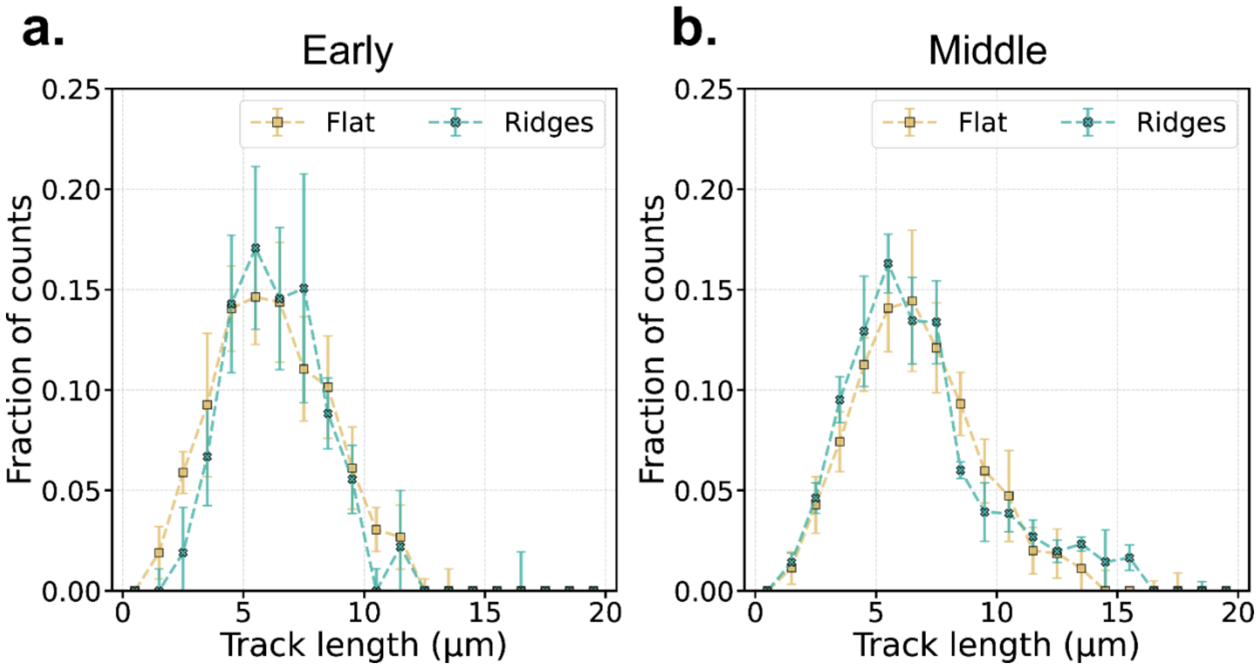

Suppl. Fig. 3. Non-cumulative histograms of track lengths, where track length is defined as the total distance traveled by a particle calculated by summing frame-to-frame displacements, at the (a) early and (b) middle developmental stages. Distributions are calculated using N = 20 bins. Error bars represent the interquartile range (IQR) of the probability density function (PDF) at each bin across all videos for each condition, and markers indicate the corresponding median values. The early stage includes 17 flat and 9 ridge movies; the middle stage includes 21 flat and 6 ridge movies.

2. Videos

Suppl. Movies 1-2. Time-lapse fluorescence recordings of actin dynamics in primary cortical neurons cultured on (1) a flat surface at DIV7 and (2) a nanoridged surface at DIV8, where DIV refers to days in vitro after plating. In the nanoridged condition, the nanoridge orientation is 68.2° relative to the x-axis of the image frame. Scale bar: 50 μm. The timestamp shows elapsed time in minutes and seconds.

3. Table

Statistical analysis of actin dynamics metrics. Results are shown for (i) the number of actin tracks normalized by cell area and movie duration, and (ii) mean track recurrence. The tables include the specific statistical tests performed along with corresponding p-values.
